# Anesthetic management of a patient with a giant ovarian tumor containing 83 l of fluid

**DOI:** 10.1186/2193-1801-2-487

**Published:** 2013-09-26

**Authors:** Keiko Bamba, Tatsunori Watanabe, Tatsuro Kohno

**Affiliations:** Division of Anesthesiology, Niigata University Graduate School of Medical and Dental Sciences, 1-757 Asahimachi, Chuo ku, 951-8510 Niigata, Japan

**Keywords:** Giant ovarian tumor, General anesthesia, Intubation under conscious sedation, Spontaneous respiration, Slow aspiration

## Abstract

We report the anesthetic management of a patient scheduled for tumor resection with a giant ovarian tumor containing 83 l of fluid. A 59-year-old woman [height 154 cm; weight 146 kg (ideal: 52 kg)] with a giant ovarian tumor was scheduled for tumor resection. Her preoperative abdominal circumference was 194 cm, which made supine positioning difficult. The thoracoabdominal computed tomography revealed a right giant cystic ovarian tumor with an estimated mass of 100 kg. Evidence of malignant tumor was not observed. In the operation room, she was intubated using a video laryngoscope (Airway Scope®, Hoya, Tokyo, Japan) in a semirecumbent position under conscious sedation. Following general anesthesia, the tumor fluid was gradually aspirated at a rate of 500 ml/min, and during this procedure, spontaneous respiration was preserved with pressure support ventilation. After the fluid was drained, the tumor was resected in a supine position. There were no major perioperative complications in hemodynamic and respiratory status, such as supine hypotensive syndrome or re-expansion pulmonary edema. Her weight decreased to 50 kg postoperatively. Maintenance of spontaneous respiration and slow aspiration of the tumor fluid prevented respiratory and hemodynamic failure and resulted in safe anesthesia management during giant ovarian tumor resection.

## Introduction

Patients with giant ovarian tumors are considered at greater risk of perioperative complications and require meticulous anesthetic management. Before the tumor is removed, supine hypotensive syndrome and ventilatory failure can be induced by compression of the tumor. A rapid decrease in thoracic pressure after removal of giant ovarian tumors can cause hemodynamic collapse and re-expansion pulmonary edema. If the ovarian tumor is benign and cystic, it has been previously reported that maintaining spontaneous respiration and slow aspiration of the cystic tumor fluid before surgical resection were effective to prevent such complications (Miyawaki et al. 2000; Nishiyama & Hanaoka 1997). However, the fluid volume of tumors in previous reports was less than 50 l. We report the anesthetic management of a patient scheduled for tumor resection with a giant ovarian tumor containing 83 l of fluid.

## Case report

**Figure 1:**
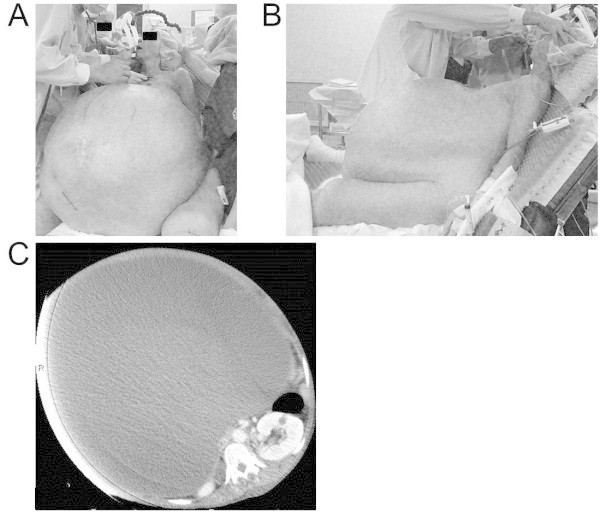
**Preoperative frontal (A) and lateral (B) view of the patient showed remarkably distended abdomen with dilated subcutaneous veins.** The abdominal computed tomography **(C)** of showed a huge cystic mass.

A 59-year-old woman [height 154 cm; weight 146 kg (ideal: 52 kg)] with a giant ovarian tumor was scheduled for tumor resection. She had no significant medical history. Her preoperative abdominal circumference was 194 cm, and she could not assume a supine position. She was assessed as grade 5 according to the Hugh-Jones classification for assessment of breathlessness. Transcutaneous oxygen saturation on room air was 94–96% in a semirecumbent position. Preoperative pulmonary function tests indicated restrictive impairment [vital capacity (VC): 1910 ml (%VC: 77.3%); forced expiratory volume in 1 second (FEV_1.0_): 1440 ml (%FEV_1.0_: 75.4%)]. Tumor markers were within normal ranges. Chest X-ray revealed elevation of the bilateral diaphragm, and thoracoabdominal computed tomography revealed a right giant cystic ovarian tumor with an estimated mass of 100 kg. Evidence of malignant tumor, atelectasis, and pleural effusion was not observed (Figure 1).

To facilitate precise hemodynamic control, standard American Society of Anesthesiologists monitors were applied and a right radial arterial line was inserted to measure arterial blood pressure; cardiac output (CO) and stroke volume variation (SVV) were measured with a FloTrac™/Vigileo™ system (Edwards Lifesciences, Tokyo, Japan). For intubation under conscious sedation, we sprayed topical 4% lidocaine from the posterior pharynx to the glottis while oxygenating with a mask at 10 l/min over 10 min. After the administration of 1 mg midazolam and 50 μg fentanyl, the trachea was intubated using the Airway Scope® (Hoya, Tokyo, Japan) in a semirecumbent position. Anaesthesia was then induced with 80 mg propofol, and maintained with 1.5% sevoflurane and 0.2 mg/h remifentanil (0.06 μg·kg^-1^·min^-1^ for 60 kg body weight). Spontaneous respiration was maintained and pressure support ventilation consisted of positive end-expiratory pressure (PEEP) set at 5 cmH_2_O with pressure support set at 17 cmH_2_O above PEEP. A catheter was placed in the right internal jugular vein under ultrasound guidance to monitor central venous pressure (CVP) and to administer inotropic agents.

Surgery commenced in the semirecumbent position to prevent supine hypotensive syndrome and respiratory failure. The incision site was infiltrated with 20 ml of 0.75% ropivacaine, a double-balloon suction catheter (SAND®, Hakko, Nagano, Japan) was inserted into the tumor, and the tumor fluid was extracted at a rate of 500 ml/min. We administered 12–24 mg/h dopamine with a transfusion because arterial blood pressure gradually decreased during fluid extraction. Draining the tumor fluid improved respiratory compliance; therefore, we decreased the pressure support to 11 cmH_2_O. Aspiration of the tumor fluid took 160 min with a total volume of 83 l. The patient was then moved into a supine position. Residual tumor resection and bilateral salpingo-oophorectomy were performed. Spontaneous respiration ceased when we increased the dose of remifentanil to 0.6 mg/h; therefore, we switched from pressure-supported ventilation to pressure-cycled ventilation. The total operation time was 667 min and the anesthesia time was 811 min. Postoperative analgesia included a continuous infusion of 40 μg/h fentanyl.

The patient was extubated on postoperative day 1 and her respiratory status was good thereafter. Her weight decreased to 50 kg postoperatively. The final pathology diagnosis of the extracted tumor was right benign ovarian cyst.

## Discussion

We present the anesthetic management without major complications of a patient with a giant ovarian tumor with a fluid volume of 83 l.

Slow aspiration of cystic tumor fluid before ovarian resection cannot be done when the tumor is entirely solid or if malignancy is suspected, because a puncture in such cases could cause dissemination of malignant cells. In our case, the tumor was cystic with no evidence of malignancy on tumor markers and thoracoabdominal CT, which allowed us to proceed with fluid aspiration as our initial step in tumor removal.

We selected general anesthesia during the fluid-extraction procedure. Because of the giant tumor size, there was a high risk of hemodynamic collapse and re-expansion pulmonary edema even after fluid extraction and these complications would have made emergency airway maintenance difficult. There have been reports of similar cases performed using epidural anesthesia (Miyawaki et al. 2000; Nishiyama & Hanaoka 1997) for the fluid-extraction procedure. However, we avoided epidural anesthesia in this case for the following reasons: 1) high risk of epidural hematoma formation because of dilatation of the epidural venous plexus; 2) potential technical difficulties due to increased internal epidural pressure; and 3) potential hemodynamic instability associated with administration of local anesthetics into the epidural space.

We intubated our patient using the Airway Scope in a semirecumbent position under conscious sedation because she was considered to have a fully dilated abdomen and the absence of spontaneous respirations would cause ventilatory failure. In addition, the semirecumbent position effectively prevented hypoxemia due to increased compression of the lungs as well as supine hypotensive syndrome. The Airway Scope is useful in a variety of positions (Komasawa et al. 2010) and, as in previous reports, (Suzuki et al. 2009) we were able to perform successful tracheal intubation using the Airway Scope in a semirecumbent position. We elected to maintain spontaneous respirations because controlled ventilation would have been difficult owing to elevated intra-abdominal pressure during tumor fluid extraction. To maintain spontaneous respiration, analgesia was performed using local anesthetic infiltration and no muscle relaxants were used. Maintaining spontaneous respiration with pressure-supported ventilation and PEEP prevented hypoxemia and hypercapnia. As the abdominal pressure decreased after tumor fluid extraction, controlled ventilation could be easily achieved.

We believed that gradual fluid extraction would effectively prevent re-expansion pulmonary edema. Re-expansion pulmonary edema is a non-cardiogenic pulmonary edema caused by rapid expansion of the lungs after long-term collapse and the risk factors include lung collapse over a period of 3 or more days, or evacuation volume of 2000 ml or more. The onset is rapid, usually within 1 h after re-expansion of the lung, (Sohara 2008) and there is no consensus with respect to an ideal extraction rate for preventing re-expansion pulmonary edema in a patient with a giant cystic ovarian tumor. In previous reports, extraction rates of 44.3 l in 2 h (22.2 l/h) (Nishiyama & Hanaoka 1997) or 11 l in 20 min (33 l/h) (Miyawaki et al. 2000) prevented re-expansion pulmonary edema. In our case, the drainage rate of 500 ml/min (30 l/h) did not cause re-expansion pulmonary edema. We believed that this rate was reasonable based on previous reports. Also, although the chest X-ray in our patient showed an elevation in the bilateral diaphragm, the CT images indicated no atelectasis, a result of anteroposterior and lateral expansion of the thoracic cavity. The absence of preoperative lung collapse was likely another factor preventing re-expansion pulmonary edema in this case.

The gradual removal of cystic tumor fluid, maintenance of spontaneous respiration during fluid extraction, and monitoring of CO, SVV and CVP provided stable hemodynamic management during surgery. Local anesthetic infiltration minimized the administration of opioids and allowed the patient to maintain spontaneous respiration. The rapid removal of fluid from a giant cystic ovarian tumor can lead to unexpected redistribution of blood and increasing intrapleural pressure by controlled ventilation decreases venous return. These procedures cause hypotension by preload reduction, and avoiding these procedures maintained stable hemodynamics in our patient. Monitoring of CO, SVV and CVP was useful to determine the appropriate dopamine dose and transfusion.

We report the anesthesia of a patient with a giant ovarian tumor with a tumor fluid volume of 83 l. Maintenance of spontaneous respiration and slow aspiration of the tumor fluid prevented respiratory and hemodynamic failure and resulted in safe anesthetic management during giant ovarian tumor resection.

## Consent

Written informed consent was obtained from the patient for publication of this case report.
